# Visit-to-visit HbA1c variability and subsequent renal function decline in older adults with type 2 diabetes

**DOI:** 10.3389/fendo.2026.1866032

**Published:** 2026-06-23

**Authors:** Tianchi Hu, Xueqin Chen, Yi Zhou, Lin Lin, Wanzhang Li, Yanjing Fan

**Affiliations:** 1 Department of Endocrinology, Xiamen Hospital, Beijing University of Chinese Medicine (Xiamen Hospital of Traditional Chinese Medicine), Xiamen, China; 2 Department of Traditional Chinese Medicine, The First Affiliated Hospital of Xiamen University, Xiamen, China

**Keywords:** chronic kidney disease, HbA1c variability, older adults, renal function decline, type 2 diabetes

## Abstract

**Background:**

Visit-to-visit HbA1c variability may capture glycemic instability beyond mean HbA1c. We assessed its association with subsequent renal function decline in older Chinese adults with type 2 diabetes.

**Methods:**

In a single-center prospective landmark cohort, we enrolled adults with type 2 diabetes who were aged ≥65 years. Index diabetes-care encounters were prospectively identified in 2015–2019. HbA1c variability was calculated from at least three HbA1c measurements collected during a fixed 24-month exposure window, and participants were followed from the 24-month landmark. The primary exposure was the HbA1c coefficient of variation (HbA1c-CV). The primary outcome was sustained renal function decline after a landmark, defined as confirmed ≥20% eGFR decline, eGFR <15 mL/min/1.73 m^2^, or kidney replacement therapy. Multivariable Cox models adjusted for mean HbA1c and baseline covariates.

**Results:**

The cohort included 630 participants (mean age 72.9 years; 52.7% men). During a median of 4.6 years of follow-up, 247 primary renal events occurred. Each 5% absolute increase in HbA1c-CV was associated with higher renal decline risk (HR 1.19, 95% CI 1.08–1.31; *p* < 0.001). Compared with the lowest tertile, the highest tertile had a higher risk (HR 1.58, 95% CI 1.18–2.11; *p* = 0.002). Results were directionally consistent in competing-risk and lagged analyses.

**Conclusions:**

Greater visit-to-visit HbA1c variability was independently associated with subsequent renal function decline. HbA1c variability may represent a pragmatic prognostic signal; however, incremental predictive value beyond established kidney markers was not tested in this study.

## Introduction

The clinical intersection of type 2 diabetes, chronic kidney disease, multimorbidity, and aging has become a defining challenge for contemporary medicine, particularly in China, where diabetes prevalence, longevity, and the absolute burden of diabetic complications are all rising (1). Kidney disease remains one of the most consequential complications because it accelerates cardiovascular events, disability, treatment complexity, hospitalization, and mortality, while also narrowing therapeutic options in later life (2, 3). In older adults, these risks unfold against a background of frailty, cognitive vulnerability, polypharmacy, and competing priorities that make glycemic targets less uniform and more individualized than in younger populations (4, 5). Recent reviews focused on older adults with diabetes have further emphasized that kidney risk and cardiovascular risk are tightly linked and that management decisions in this age group should integrate prognosis, functional reserve, and treatment burden (6–8).

Although current diabetes and kidney guidelines appropriately emphasize eGFR, albuminuria, blood pressure, and the use of kidney-protective therapies, routine outpatient management still gives substantial weight to the latest or mean HbA1c value (2, 3, 9). Mean HbA1c summarizes average glycemic exposure without capturing instability in control over time, whereas visit-to-visit HbA1c variability may reflect fluctuations in adherence, therapeutic intensification or deintensification, intercurrent illness, nutritional change, and underlying biological vulnerability (10–13). These dynamics may be especially relevant in older adults, whose diabetes care often involves medication simplification, recurrent hospitalization, variable self-management capacity, and more heterogeneous metabolic trajectories than those seen in middle-aged clinic populations (4, 5, 8). Accordingly, two patients with similar mean HbA1c may still differ meaningfully in long-term glycemic stability and, potentially, in renal risk.

A growing body of observational literature suggests that higher long-term HbA1c variability is associated with adverse renal and microvascular outcomes beyond average glycemia (10–16). Recent systematic reviews, meta-analyses, and narrative reviews have reported positive associations between HbA1c variability and nephropathy progression, broader diabetes complications, and mortality outcomes (10–13). In Chinese adults with type 2 diabetes, a multicenter analysis found that a higher HbA1c variability score was linked to faster kidney function decline (14). In a large chronic kidney disease (CKD) cohort, greater glycemic variability was associated with progression to end-stage kidney disease, and a *post hoc* analysis of the CREDENCE trial similarly suggested that HbA1c variability carries prognostic information in patients with type 2 diabetes and established CKD (15, 16). At the same time, this literature remains methodologically heterogeneous, particularly in how exposure windows, measurement density, and outcome follow-up are aligned (10–16). The gap remains clinically relevant because the population burden of diabetic CKD is still substantial (17).

However, important gaps remain for older adults because many prior cohorts enrolled broader adult populations, used differing HbA1c variability metrics, or allowed partial overlap between exposure assessment and renal outcome follow-up (10, 14, 18–21). In this context, a fixed landmark design is useful because HbA1c variability is quantified before outcome accrual, which helps reduce immortal time bias and reverse causation when renal decline itself may alter monitoring or treatment intensity.

HbA1c is already measured repeatedly in routine care, so a reproducible variability metric could, in principle, be explored as a low-cost adjunctive risk-review signal, provided that its association with kidney outcomes remains robust after accounting for mean HbA1c, baseline renal function, albuminuria, comorbidity burden, and kidney-protective medications (3–5, 9). We therefore designed a prospective landmark cohort study in which HbA1c variability was measured during a fixed 24-month pre-outcome window, and renal events were prospectively ascertained after the landmark time zero. The objective was to determine whether higher visit-to-visit HbA1c variability was independently associated with subsequent renal function decline in older adults with type 2 diabetes.

## Methods

### Study design and setting

This study was designed as a single-center prospective landmark cohort study using prospectively collected routine clinical, laboratory, pharmacy, and encounter data from the Xiamen Hospital, Beijing University of Chinese Medicine (Xiamen Hospital of Traditional Chinese Medicine). Eligible participants were enrolled at qualifying index diabetes-care encounters that occurred between 1 January 2015 and 30 June 2019. Index encounter was defined *a priori*, operationalized as the first qualifying outpatient diabetes-care visit from which a complete 24-month exposure began and was documented in the study data. For each participant, a fixed 24-month exposure window was defined after the index encounter to quantify visit-to-visit HbA1c variability, and the landmark time zero was set exactly 24 months after the index; renal outcome follow-up began the day after that landmark and continued through 30 June 2025. The landmark structure was prespecified to separate exposure assessment from event accrual and thereby reduce immortal time bias and reverse causation. Renal function surveillance across the study used creatinine-based eGFR estimation anchored to the 2021 CKD-EPI framework (22). Study reporting was aligned with the STROBE reporting frameworks (23).

### Participants and landmark eligibility

Participants were required to be aged 65 years or older at landmark, to have type 2 diabetes documented by an endocrinologist diagnosis, ICD coding, or antidiabetic treatment history; and to have at least three HbA1c measurements during the 24-month exposure window spanning at least 12 months. Additional eligibility criteria were landmark eGFR of at least 30 mL/min/1.73 m^2^ and at least one creatinine-based eGFR measurement within 6 months before the landmark. Post-landmark creatinine measurements were not used to determine cohort entry. Instead, all landmark-eligible participants contributed follow-up until the last valid creatinine value, and a second measurement separated by at least 90 days was required only to confirm a sustained decline event. Exclusion criteria were type 1, gestational, pancreatic, or secondary diabetes; prior kidney replacement therapy, transplantation, kidney failure, or landmark eGFR below 30 mL/min/1.73 m^2^; renal decline, death, or transfer out before landmark; unresolved acute kidney injury within 90 days before landmark; conditions likely to distort HbA1c interpretation during the exposure window, including recent transfusion, hemolytic anemia, erythropoietin initiation, or dialysis; active malignancy with expected survival under 12 months; and key-variable quality-control failure.

### Exposure definition and laboratory handling

The primary exposure was visit-to-visit HbA1c coefficient of variation (HbA1c-CV), calculated within person during the 24-month exposure window as the standard deviation divided by the mean HbA1c multiplied by 100. HbA1c-CV was analyzed both by tertiles defined from the analytic cohort distribution and continuously per 5% absolute increase. Mean HbA1c over the same exposure window was treated as a prespecified covariate so that the primary contrast reflected variability beyond average glycemic exposure. Secondary variability metrics were HbA1c standard deviation, variability independent of the mean, average real variability, and the HbA1c variability score based on successive changes of at least 0.5%. Inpatient HbA1c values were retained in the primary analysis to capture real-world care-process variability, but a sensitivity analysis excluded them. Serum creatinine was converted to eGFR with the 2021 Chronic Kidney Disease Epidemiology Collaboration creatinine equation (22).

HbA1c values were obtained during routine outpatient and inpatient care, rather than at protocol-mandated intervals; monitoring intensity was therefore addressed by adjustment for HbA1c measurement count and by the monitoring-intensity weighted sensitivity analysis. Within the available routine-care laboratory records, HbA1c results were reported in NGSP (%) and IFCC (mmol/mol) units under the hospital central laboratory quality-control system; however, the analytic dataset did not include a separate patient-level variable documenting assay-platform changes, and this was therefore considered in the limitations rather than adjusted analytically (24). Serum creatinine measurements were likewise obtained during routine care, and all valid post-landmark creatinine values were used for outcome ascertainment with a confirmation requirement of at least 90 days.

### Outcome definitions and follow-up

The primary endpoint was time from landmark to first sustained renal function decline, defined as a confirmed decrease in eGFR of at least 20% from the landmark value on two measurements separated by at least 90 days, eGFR below 15 mL/min/1.73 m^2^, or kidney replacement therapy. Key secondary endpoints were sustained decline of at least 30% in eGFR or kidney failure, rapid kidney function decline defined as annual eGFR slope steeper than −3.0 mL/min/1.73 m^2^ per year, and incident CKD G3+ among participants with landmark eGFR at least 60 mL/min/1.73 m^2^. Definitions were selected to remain clinically interpretable and broadly consistent with contemporary kidney and diabetes guidance, emphasizing reproducible eGFR decline, CKD staging, and outcome confirmation (2, 3, 9). Participants were censored at the first renal event, death before renal event, transfer out, last valid creatinine measurement, or administrative end of follow-up on 30 June 2025, whichever came first. Because death could preclude observation of renal decline in this older cohort, competing-risk analyses were prespecified.

The ≥20% threshold was selected as a sensitive observational endpoint for older adults with mostly non-kidney failure baseline eGFR, and the requirement for confirmation at least 90 days apart was intended to reduce misclassification from biologic or analytic fluctuation. Because larger sustained eGFR declines have stronger validation as trial surrogate endpoints, the ≥30% eGFR decline or kidney failure endpoint was prespecified as a stricter secondary analysis (25, 26).

### Covariates, missing data, and confounding control

Covariates were measured before or at landmark and were chosen *a priori* based on subject-matter knowledge of factors plausibly related to both HbA1c variability and renal decline. Continuous covariates were defined as the measurement closest to the landmark within the preceding 180 days; the urine albumin-to-creatinine ratio used the closest value within the preceding 365 days. These included age, sex, diabetes duration, body mass index, current smoking, systolic blood pressure, cardiovascular disease, landmark eGFR, log-transformed urine albumin-to-creatinine ratio, mean HbA1c, number of HbA1c measurements during the exposure window, low-density lipoprotein cholesterol, hemoglobin, serum albumin, uric acid, and baseline use of renin–angiotensin system inhibitors, sodium–glucose cotransporter 2 inhibitors, insulin, and statins. Post-landmark treatment changes and post-landmark physiologic measurements were not entered into primary models because they may lie on the causal pathway or act as colliders. Exposure variables were not imputed. For non-exposure covariates with incomplete values, the main analytic strategy used multiple imputation by chained equations under a missing-at-random assumption, with 20 imputed datasets combined using Rubin’s rules; complete-case analysis and a monitoring-intensity weighted sensitivity analysis were used to examine the influence of selection and surveillance patterns.

Information on glucose-lowering therapy was limited to medication categories that were reliably available across the full study period; therefore, glucagon-like peptide-1 receptor agonist exposure and other non-insulin, non-SGLT2 glucose-lowering therapies were not incorporated into primary adjustment models. This limitation is relevant because GLP-1 receptor agonists may influence glycemic stability and kidney outcomes in type 2 diabetes with CKD (9, 27). Hypoglycemia episodes and hospitalizations were also not used as covariates because validated event definitions were not uniformly available in the analytic dataset; these factors are therefore addressed as potential residual confounding in the Discussion.

### Statistical analysis and power rationale

Baseline characteristics were summarized overall and by HbA1c-CV tertile using mean ± standard deviation (SD), median [interquartile range, IQR], or number (percentage) as appropriate. The primary association between HbA1c-CV and time to renal decline was estimated with Cox proportional hazards models from landmark time zero. Model 1 adjusted for age, sex, and HbA1c measurement count; model 2 additionally adjusted for mean HbA1c, diabetes duration, landmark eGFR, and log urine albumin-to-creatinine ratio; model 3 additionally adjusted for body mass index, systolic blood pressure, smoking, cardiovascular disease, hemoglobin, albumin, uric acid, and use of renin–angiotensin system inhibitors, sodium–glucose cotransporter 2 inhibitors, insulin, and statins. Restricted cubic splines with three knots at the 10th, 50th, and 90th percentiles were used as an exploratory functional-form check, centered at the cohort median HbA1c-CV. Proportional hazards, influential observations, functional form, and collinearity were examined using Schoenfeld residuals, dfbeta statistics, spline-based residual inspection, and variance inflation factors. Sensitivity analyses included Fine–Gray competing-risk regression, stricter renal endpoints, exclusion of events within 6 and 12 months after landmark, exclusion of inpatient HbA1c values, and inverse-probability weighting for monitoring intensity using baseline demographic, kidney, glycemic, and visit-frequency variables. Benjamini–Hochberg false discovery rate adjustment was applied within the prespecified secondary/sensitivity family and separately within the subgroup family. Prespecified subgroup analyses examined age, sex, landmark eGFR category, albuminuria category, and insulin use. The analysis sample was determined by prospectively applied eligibility criteria; based on the variables explicitly listed for model 3, the locked primary event count of 247 yielded approximately 12.3 events per modeled degree of freedom for the tertile model and 13.0 for the continuous model. Analyses were performed in R version 4.4 using survival, rms, cmprsk, and mice packages.

## Results

### Cohort assembly and follow-up

**Figure 1:**
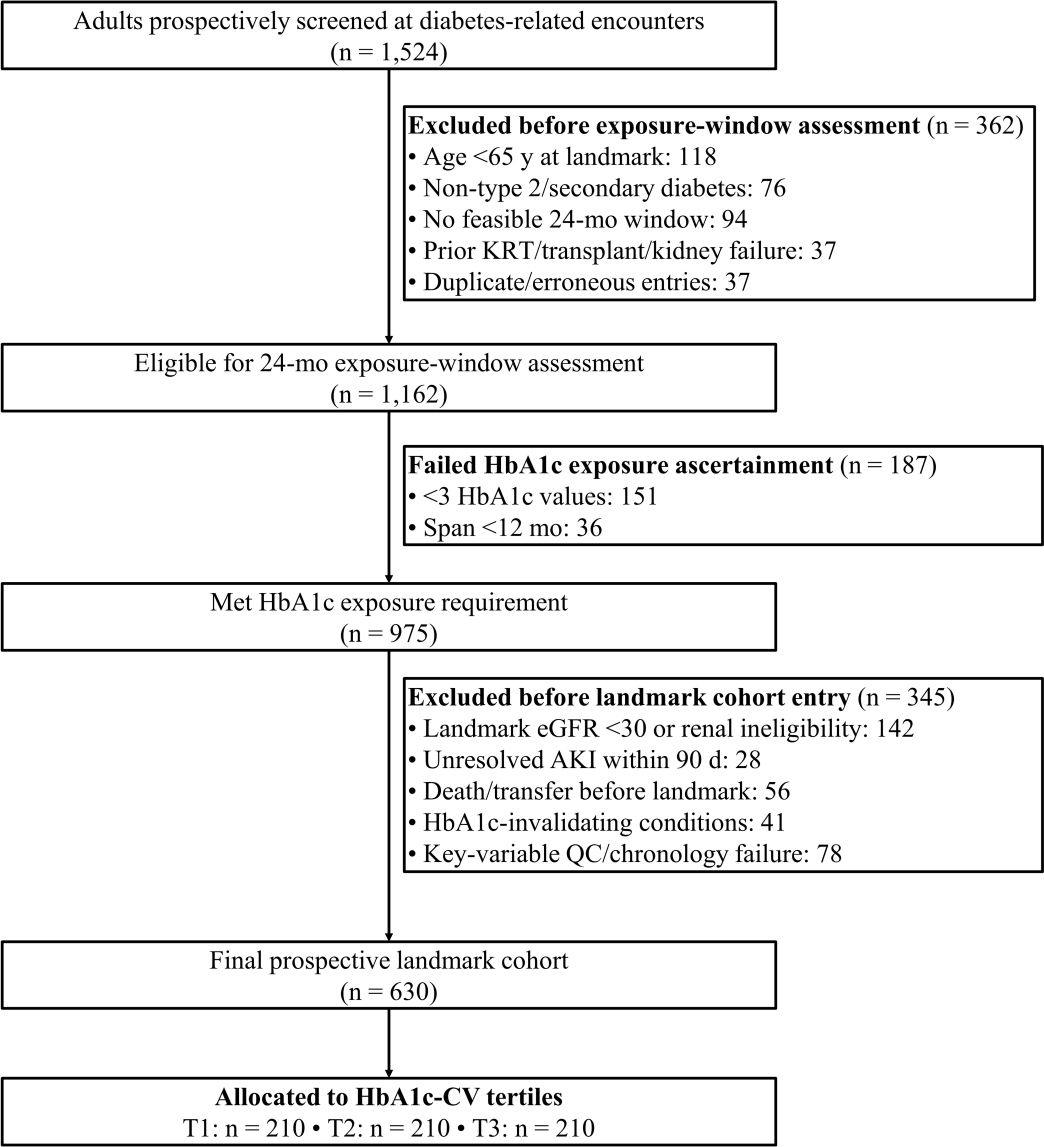
Cohort assembly in the prospective landmark design. The analytic cohort comprised 630 adults aged 65 years or older at the 24-month landmark.

A total of 1,524 adults presenting for diabetes-related encounters were prospectively screened. After exclusion of 362 participants before exposure-window assessment, including 37 duplicate or erroneous entries, 1,162 remained eligible for 24-month exposure-window assessment, and 975 met the HbA1c measurement requirement (**Figure 1**). After exclusion of patients with pre-landmark renal ineligibility, unresolved acute kidney injury, death or transfer before time zero, HbA1c-invalidating conditions, and key-variable quality-control/chronology failures, 630 participants entered the analytic cohort. Median post-landmark follow-up was 4.6 years [3.8–6.1], providing 2,898 person-years of observation.

### Baseline characteristics by HbA1c variability tertile

With the cohort defined, we next compared baseline characteristics across HbA1c-CV tertiles (**Table 1**). Overall mean age was 72.9 ± 5.5 years, 332 participants (52.7%) were men, median diabetes duration was 11.0 [7.0–16.0] years, mean HbA1c was 7.8% ± 1.1% (62 ± 12 mmol/mol), and mean landmark eGFR was 71.6 ± 18.4 mL/min/1.73 m^2^. Higher variability was accompanied by a more adverse clinical profile: across tertiles, diabetes duration increased from 9.0 to 13.0 years, mean HbA1c from 7.3% to 8.3%, median HbA1c measurement count from 4 to 6, UACR ≥30 mg/g from 40.0% to 59.5%, landmark eGFR declined from 75.0 to 67.6 mL/min/1.73 m^2^, and insulin use increased from 31.9% to 51.4% (**Table 1**).

**Table 1:** Baseline characteristics by HbA1c coefficient-of-variation tertile.

Characteristic	Tertile 1	Tertile 2	Tertile 3	*P*-value
Participants, *n*	210	210	210	–
Age, years	72.5 ± 5.2	72.8 ± 5.4	73.3 ± 5.8	0.21
Men, *n* (%)	108 (51.4)	112 (53.3)	112 (53.3)	0.90
Diabetes duration, years	9.0 [6.0, 14.0]	11.0 [7.0, 16.0]	13.0 [9.0, 18.0]	<0.001
Body mass index, kg/m^2^	24.6 ± 3.1	24.8 ± 3.1	25.2 ± 3.4	0.15
Current smoking, *n* (%)	35 (16.7)	39 (18.6)	43 (20.5)	0.62
Systolic blood pressure, mmHg	129.8 ± 13.9	132.7 ± 14.2	136.8 ± 15.6	<0.001
Cardiovascular disease, *n* (%)	57 (27.1)	65 (31.0)	75 (35.7)	0.12
Mean HbA1c, %	7.3 ± 0.9	7.8 ± 1.0	8.3 ± 1.1	<0.001
Mean HbA1c, mmol/mol	56 ± 10	62 ± 11	67 ± 12	<0.001
HbA1c measurements in the exposure window	4 [3, 4]	5 [4, 6]	6 [5, 7]	<0.001
Landmark eGFR, mL/min/1.73 m^2^	75.0 ± 17.5	72.3 ± 18.0	67.6 ± 18.8	<0.001
UACR, mg/g	24 [10, 64]	35 [14, 110]	58 [23, 171]	<0.001
UACR ≥30 mg/g, *n* (%)	84 (40.0)	102 (48.6)	125 (59.5)	<0.001
LDL-C, mmol/L	2.34 ± 0.74	2.41 ± 0.76	2.52 ± 0.82	0.08
Hemoglobin, g/L	132 ± 15	129 ± 16	126 ± 17	<0.001
Serum albumin, g/L	42.4 ± 3.6	42.0 ± 3.7	41.3 ± 4.0	0.01
Uric acid, µmol/L	343 ± 78	357 ± 84	371 ± 91	0.002
RAS inhibitor use, *n* (%)	122 (58.1)	128 (61.0)	136 (64.8)	0.34
SGLT2 inhibitor use, *n* (%)	35 (16.7)	42 (20.0)	53 (25.2)	0.07
Insulin use, *n* (%)	67 (31.9)	86 (41.0)	108 (51.4)	<0.001
Statin use, *n* (%)	129 (61.4)	138 (65.7)	146 (69.5)	0.23

Continuous variables are mean ± SD or median [IQR]; categorical variables are *n* (%).

### Crude renal outcomes and Kaplan–Meier event estimates

**Figure 2:**
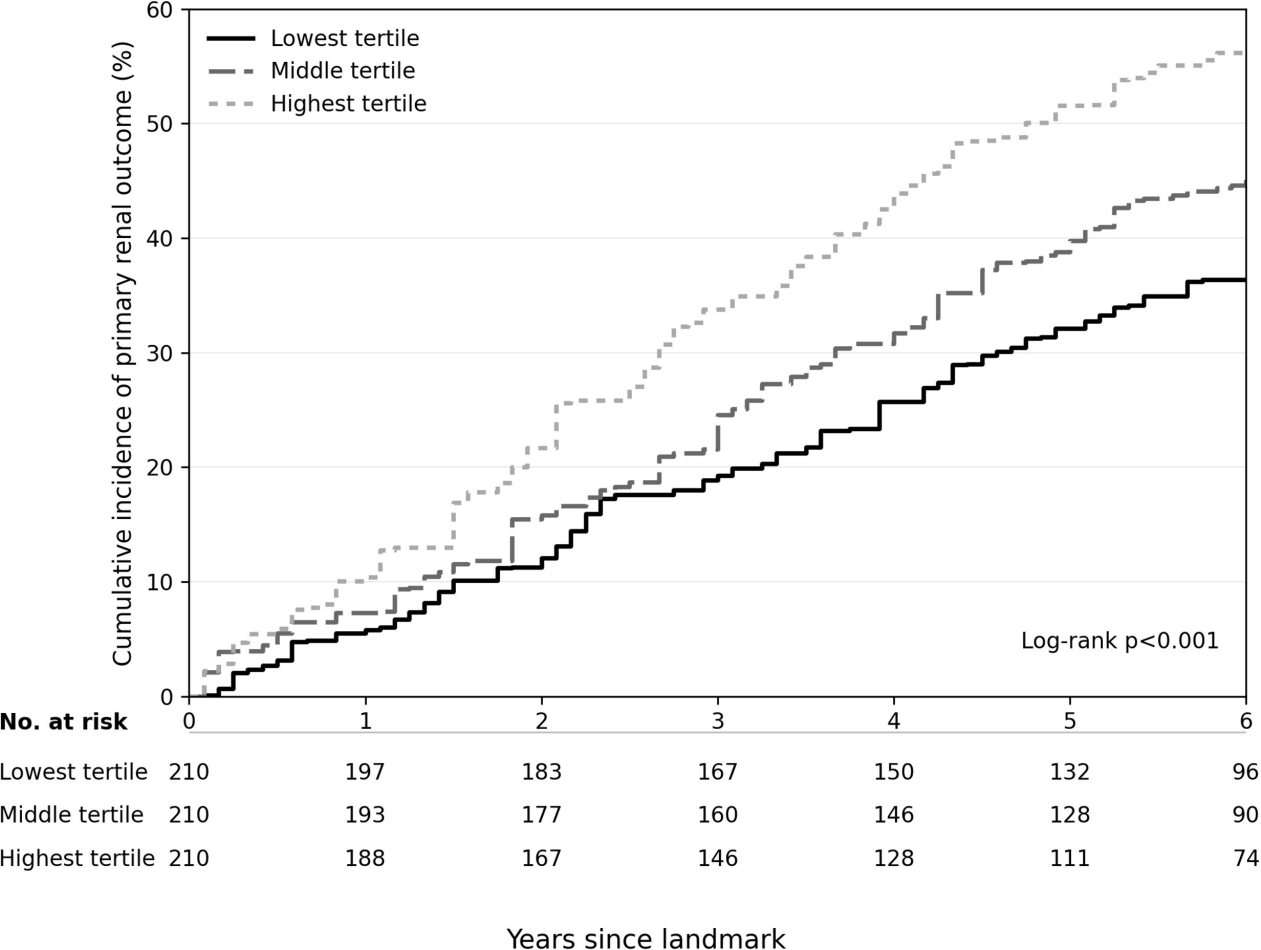
Kaplan–Meier event estimates of confirmed renal function decline after the 24-month landmark, by HbA1c-CV tertile. Progressively higher post-landmark renal risk with increasing visit-to-visit HbA1c variability. The plotted 5-year event estimates are 32.1% for tertile 1, 39.8% for tertile 2, and 51.6% for tertile 3; log-rank *p* < 0.001.

Median HbA1c-CV was 9.4% [7.0–12.7], with tertile medians of 6.3%, 9.2%, and 13.6% (**Table 2**). During follow-up, 247 participants (39.2%) developed the primary renal decline outcome, corresponding to 8.5 events per 100 person-years (95% CI 7.5–9.7) and 5-year Kaplan–Meier event estimate of 41.0% (95% CI 37.1–45.0). Event burden increased across tertiles, with event proportions of 30.0%, 37.6%, and 50.0% and incidence rates of 6.2, 8.2, and 11.5 per 100 person-years. **Figure 2** shows early and sustained curve separation; the 5-year event estimates were 32.1%, 39.8%, and 51.6%, respectively (log-rank *p* < 0.001). Similar crude gradients were seen for the ≥30% eGFR decline or kidney failure endpoint, rapid eGFR decline, and incident CKD G3 +.

**Table 2:** Crude renal outcomes and follow-up by HbA1c coefficient-of-variation tertile.

Outcome or metric	Tertile 1	Tertile 2	Tertile 3	*P*-value
HbA1c-CV, median [IQR], %	6.3 [5.5, 7.0]	9.2 [8.4, 10.1]	13.6 [12.2, 15.6]	–
Follow-up, years	4.8 [4.0, 6.4]	4.6 [3.8, 6.1]	4.5 [3.6, 5.8]	0.08
Person-years	1014.5	966.8	916.7	–
Primary renal decline, *n* (%)	63 (30.0)	79 (37.6)	105 (50.0)	<0.001
Incidence rate/100 person-years (95% CI)	6.2 (4.8–8.0)	8.2 (6.5–10.2)	11.5 (9.4–13.9)	–
5-year Kaplan–Meier event estimate, % (95% CI)	32.1 (25.7–38.7)	39.8 (33.0–46.4)	51.6 (44.5–57.8)	log-rank <0.001
≥30% eGFR decline or kidney failure, *n* (%)	46 (21.9)	61 (29.0)	80 (38.1)	<0.001
Rapid eGFR decline, *n* (%)	40 (19.0)	56 (26.7)	75 (35.7)	<0.001
Incident CKD G3+ among eGFR ≥60 at landmark, *n*/*N* (%)	25/166 (15.1)	30/154 (19.5)	41/142 (28.9)	0.006
Non-renal death before renal decline, *n* (%)	16 (7.6)	18 (8.6)	24 (11.4)	0.31

Renal decline denotes confirmed post-landmark renal function decline. Incidence rate and 95% CIs are Poisson intervals.

### Adjusted associations and dose–response pattern

**Figure 3:**
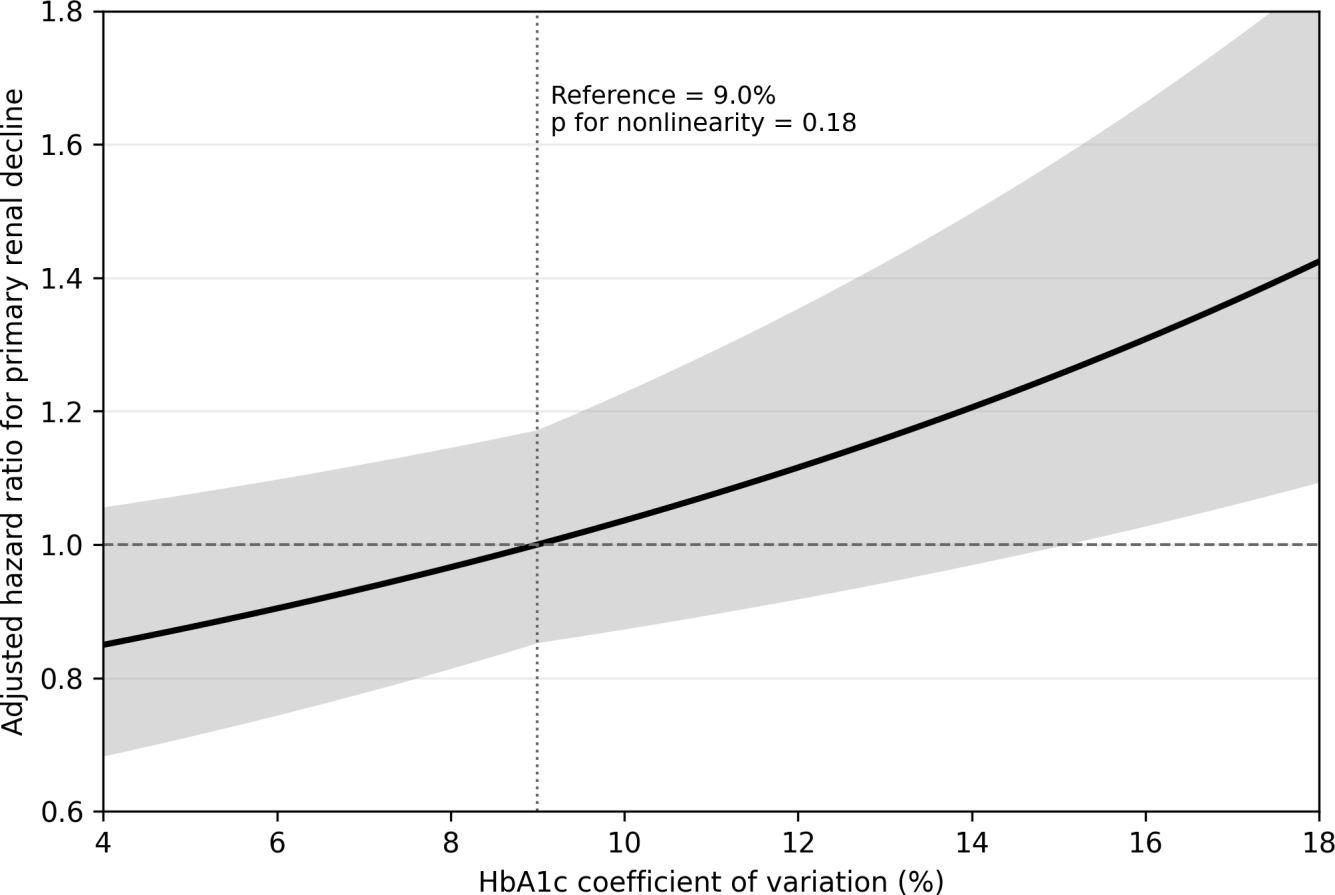
Adjusted dose–response relation between HbA1c-CV and primary renal decline. Restricted cubic spline display centered at the cohort median HbA1c-CV. The relation is largely monotonic with little evidence of non-linearity (*p* for non-linearity = 0.18). Shaded bands represent 95% confidence intervals.

In **Table 3**, the highest HbA1c-CV tertile was associated with the primary outcome in model 1 (HR 1.71, 95% CI 1.28–2.29; *p* < 0.001), and the association remained after adding mean HbA1c, diabetes duration, landmark eGFR, and albuminuria in model 2 (HR 1.61, 95% CI 1.20–2.16; *p* = 0.001) and after full adjustment in model 3 (HR 1.58, 95% CI 1.18–2.11; *p* = 0.002). The middle tertile was not significantly different from the lowest tertile in the fully adjusted model (HR 1.22, 95% CI 0.90–1.64; *p* = 0.20). When modeled continuously, each 5% absolute increase in HbA1c-CV was associated with a 19% higher hazard of renal decline (HR 1.19, 95% CI 1.08–1.31; *p* < 0.001). **Figure 3** shows a largely monotonic relation with little evidence of non-linearity (*p* for non-linearity = 0.18), supporting the prespecified linear and tertile-based interpretation.

**Table 3:** Association of HbA1c variability with renal outcomes: primary, sensitivity, and secondary analyses.

Analysis set	Contrast	HR	95% CI	*P*-value	*q*-value
Model 1	Middle vs. lowest tertile	1.25	0.94–1.68	0.12	–
Model 1	Highest vs. lowest tertile	1.71	1.28–2.29	<0.001	–
Model 1	Per 5% higher HbA1c-CV	1.22	1.12–1.34	<0.001	–
Model 2	Middle vs. lowest tertile	1.23	0.91–1.65	0.17	–
Model 2	Highest vs. lowest tertile	1.61	1.20–2.16	0.001	–
Model 2	Per 5% higher HbA1c-CV	1.20	1.09–1.32	<0.001	–
Model 3	Middle vs. lowest tertile	1.22	0.90–1.64	0.20	–
Model 3	Highest vs. lowest tertile	1.58	1.18–2.11	0.002	–
Model 3	Per 5% higher HbA1c-CV	1.19	1.08–1.31	<0.001	–
Sensitivity	Fine–Gray competing-risk model (sHR)	1.51	1.12–2.03	0.007	0.013
Sensitivity	Exclude events within 6 months	1.54	1.13–2.09	0.006	0.013
Sensitivity	Exclude events within 12 months	1.49	1.07–2.07	0.018	0.021
Sensitivity	Exclude inpatient HbA1c values	1.53	1.14–2.05	0.005	0.013
Sensitivity	Monitoring-intensity weighting	1.47	1.09–1.98	0.012	0.013
Secondary endpoint	≥30% eGFR decline or kidney failure	1.55	1.11–2.17	0.010	0.013
Secondary endpoint	Rapid eGFR decline	1.59	1.13–2.24	0.008	0.013
Alternative metric	HbA1c variability score >60% vs. ≤20%	1.34	1.06–1.70	0.015	0.021

Model 1 adjusts for age, sex, and HbA1c measurement count. Model 2 additionally adjusts for mean HbA1c, diabetes duration, landmark eGFR, and log UACR. Model 3 additionally adjusts for body mass index, systolic blood pressure, smoking, cardiovascular disease, hemoglobin, albumin, uric acid, baseline RAS inhibitor, SGLT2 inhibitor, insulin, and statin use. Global proportional hazards test *p* = 0.41; spline nonlinearity p = 0.18; no variance inflation factor >2.5.

### Robustness analyses and prespecified subgroups

**Figure 4:**
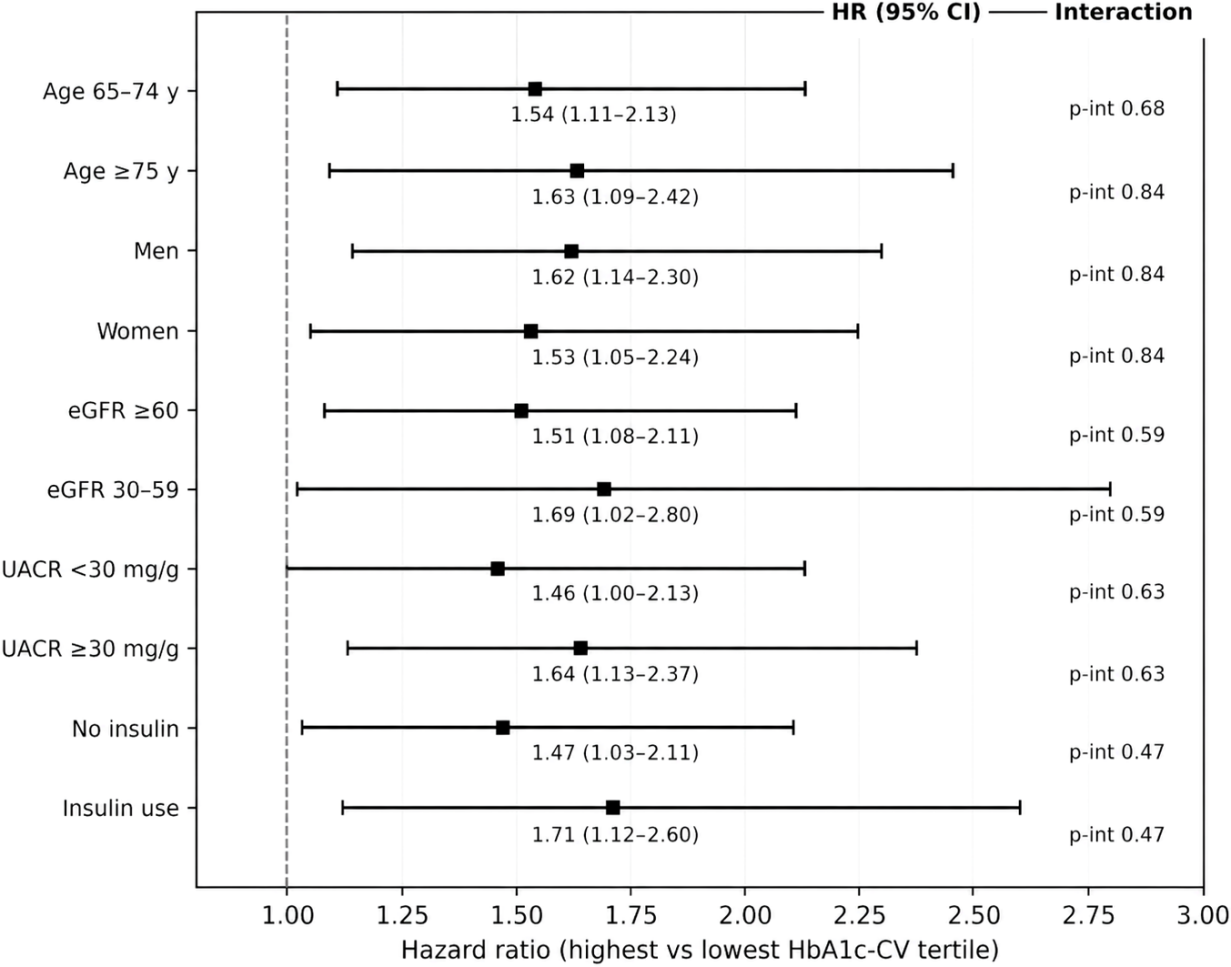
Prespecified subgroup analysis for the highest versus the lowest HbA1c-CV tertile. The forest plot showed adjusted hazard ratios across prespecified clinical strata.

As summarized in **Table 3** and **Figure 4**, the highest-versus-lowest tertile comparison remained significant for the stricter ≥30% eGFR decline or kidney failure endpoint (HR 1.55, 95% CI 1.11–2.17), rapid eGFR decline (HR 1.59, 95% CI 1.13–2.24), and the Fine–Gray competing-risk model (subdistribution HR 1.51, 95% CI 1.12–2.03). Results were also directionally consistent after excluding events within 6 months (HR 1.54, 95% CI 1.13–2.09) or 12 months (HR 1.49, 95% CI 1.07–2.07) and after monitoring-intensity weighting (HR 1.47, 95% CI 1.09–1.98). HbA1c variability score >60% versus ≤20% was similarly associated with the primary outcome (HR 1.34, 95% CI 1.06–1.70).

## Discussion

Taken together with the preceding analyses, the main finding is that higher visit-to-visit HbA1c variability was independently associated with subsequent renal function decline in older adults with type 2 diabetes. The association persisted after adjustment for mean HbA1c, baseline eGFR, albuminuria, medication use, and monitoring intensity, and it remained evident when HbA1c-CV was modeled continuously and when the renal endpoint was made more stringent. The 5-year Kaplan–Meier event estimate increased from 32.1% in the lowest HbA1c-CV tertile to 51.6% in the highest tertile. This pattern suggests that long-term glycemic instability carries information not fully captured by average HbA1c alone (10, 11, 28).

At a biological level, repeated oscillation between hyperglycemia and lower glucose exposure has been linked in prior mechanistic and review literature to oxidative stress, endothelial dysfunction, inflammatory signaling, and microvascular injury (13). In routine practice, however, HbA1c variability is also a care-process marker: it may capture intermittent adherence, medication changes, hospitalization, nutritional instability, or heterogeneous physiologic reserve, all of which are especially relevant in older adults with multimorbidity and polypharmacy (4, 5, 8). It may also reflect measurement-process factors, including inpatient testing, anemia-related distortion of HbA1c, and any unreported assay-platform changes. The prospective landmark design reduces the likelihood that early renal decline directly inflated the exposure measurement, but it does not eliminate the possibility that higher HbA1c variability identified patients whose frailty, treatment complexity, or occult illness also increased renal risk. Accordingly, the present findings support HbA1c variability as a prognostic signal rather than proof that variability itself is the proximate cause of kidney decline. In the present cohort of older adults with long-standing type 2 diabetes and suboptimal mean glycemic control, high HbA1c variability may therefore signal not only biological instability but also adherence barriers, therapeutic inertia, multimorbidity, nutritional instability, and fragmentation in outpatient diabetes management (4, 5, 8). Current smoking was numerically more common in higher HbA1c-CV tertiles; although this gradient was not statistically significant, smoking should be interpreted as a behavioral and vascular risk marker that may contribute to both unstable glycemic control and kidney injury (29, 30).

Meta-analyses have shown that higher HbA1c variability is associated with nephropathy progression and broader adverse outcomes in type 2 diabetes, supporting the view that glycemic instability is not explained solely by average HbA1c (10, 11, 20). The present study also aligns with the Chinese multicenter analysis by Zhou and colleagues, in which a higher HbA1c variability score was associated with faster kidney function decline (14). Beyond China, a CKD cohort study found that glycemic variability was associated with progression to end-stage kidney disease, and *post hoc* analysis from CREDENCE extended the association to major renal outcomes in patients with type 2 diabetes and established CKD (15, 16). Our study adds to that literature by focusing specifically on older Chinese adults and by separating HbA1c exposure ascertainment from renal event accrual with a fixed landmark, thereby addressing a design weakness that has limited causal interpretation in some prior observational work. More recent routine-care data from Stockholm similarly linked long-term HbA1c variability with CKD progression, acute kidney injury, and worsening albuminuria, whereas Asian cohort studies using HbA1c-SD, HbA1c-CV, HbA1c variability score, or HbA1c-AUC have reported broadly comparable but not identical associations with diabetic kidney disease or renal function decline (18–21). Differences among studies are likely explained by age structure, baseline CKD burden, measurement density, choice of variability metric, and whether renal outcomes were defined by albuminuria, eGFR slope, sustained eGFR decline, or kidney failure (10, 18–21). Our fixed 24-month exposure window and post-landmark outcome ascertainment were intended to minimize exposure–outcome overlap, a limitation that can complicate interpretation when HbA1c measurements after early kidney decline contribute to the variability metric.

From a clinical perspective, the immediate implication is not that HbA1c variability should replace established kidney risk markers, but that it may complement them. Clinicians should therefore view higher variability as a cue to review adherence, medication changes, hypoglycemia risk, nutrition, and intercurrent illness, not as a stand-alone reason to intensify glucose-lowering therapy (3–5, 9). Current kidney and diabetes guidance already prioritizes eGFR, albuminuria, blood pressure control, and cardiorenal-protective therapy (2, 3, 9). Within that framework, an automatically calculated HbA1c-CV or HbA1c variability score could be explored as an adjunctive review signal for kidney surveillance, albuminuria testing, medication reconciliation, and treatment-plan stability in older patients, although this study did not test incremental predictive performance beyond established kidney risk markers (3–5, 9). This interpretation is especially relevant in older adults, for whom glycemic management is individualized and the clinical question is often how to balance risk recognition against overtreatment rather than how to intensify therapy indiscriminately (4, 5, 8). However, its practical use should remain risk-review-oriented until prediction studies determine whether adding HbA1c variability improves C-statistics, net reclassification improvement, calibration, or decision-curve performance beyond eGFR, albuminuria, blood pressure, and established clinical risk factors.

As in any single-center observational study, residual confounding cannot be excluded, particularly from frailty, socioeconomic factors, dietary instability, hypoglycemia burden, and changes in treatment after the landmark. The landmark design intentionally restricts inference to patients who survived and remained under observation through the 24-month exposure window. Selection depended on repeated HbA1c and creatinine testing, so included patients may have differed systematically from less-monitored patients despite the monitoring-intensity analyses. HbA1c can be influenced by anemia, altered erythrocyte turnover, and advanced kidney disease, which may introduce exposure misclassification in older adults even after excluding major pre-landmark conditions that clearly invalidate HbA1c interpretation (3, 9). The primary endpoint used a sensitive ≥20% sustained eGFR decline threshold, although the persistence of the association for the stricter ≥30% endpoint argues against the findings being driven solely by that choice. Finally, because the study used a single center, the results should be generalized cautiously and ideally confirmed in externally validated multicenter cohorts. Specifically, medication adherence, healthcare utilization intensity, cognitive impairment, therapeutic inertia, GLP-1 receptor agonist use, hypoglycemia burden, hospitalization, and post-landmark treatment modifications may have contributed to both HbA1c variability and kidney outcomes. The ≥20% endpoint should therefore be viewed as a sensitive clinical deterioration marker rather than as a fully validated kidney failure surrogate; the consistent ≥30% sensitivity analysis partly addresses this concern (25, 26). External validity remains limited to a single-center older Chinese outpatient cohort; multicenter validation in younger, ethnically diverse, and differently organized health system cohorts is needed before broad implementation.

In summary, greater visit-to-visit HbA1c variability was associated with a higher risk of subsequent renal function decline in older adults with type 2 diabetes in this landmark cohort. The findings support HbA1c variability as a pragmatic prognostic signal beyond mean HbA1c, but incremental predictive value and external generalizability require further evaluation.

## Data Availability

The raw data supporting the conclusions of this article will be made available by the authors, without undue reservation.
